# Effect of regional anesthesia on the postoperative delirium: A systematic review and meta-analysis of randomized controlled trials

**DOI:** 10.3389/fsurg.2022.937293

**Published:** 2022-07-26

**Authors:** Tao Li, Tiantian Dong, Yuanshan Cui, Xiangrui Meng, Zhao Dai

**Affiliations:** ^1^ Department of Anesthesiology, The Affiliated Yantai Yuhuangding Hospital of Qingdao University, Yantai, China; ^2^ Department of Urology, The Affiliated Yantai Yuhuangding Hospital of Qingdao University, Yantai, China

**Keywords:** regional anesthesia, general anesthesia, delirium, pain, meta—analysis

## Abstract

**Objective:**

Postoperative delirium (POD) starts in the recovery room and occurs up to 5 days after surgery. However, the POD guidelines issued by the European Society of Anesthesiology (ESA) suggest that the effect of regional anesthesia on POD is controversial. This meta-analysis aims to investigate whether perioperative regional anesthesia reduced the incidence of POD.

**Methods:**

Standard Published randomized controlled trails (RCTs) were searched from bibliographic databases to identify all evidence that reported regional anesthesia assessing incident delirium following diverse surgeries. The primary outcome was the incidence of POD, and the secondary outcomes were POD scores, pain scores, and emergence time. The relative risk (RR) for dichotomous outcomes and the weighted or standardized mean difference (WMD, SMD) for continuous outcomes were estimated using a random-effects model.

**Results:**

Twenty RCTs with 2110 randomized participants undergoing different surgeries were included. Meta-analysis showed that regional anesthesia was associated with less POD incidence compared to general anesthesia (total intravenous anesthesia (TIVA) or inhalation anesthesia) (relative risk (RR) = 0.62, 95% confidence interval (CI) = 0.45–0.85)). Subgroup analysis showed that the decrease in POD incidence was associated with a nerve block (0.46, 95% CI = 0.32–0.67) and regional-combined-general anesthesia (0.42, 95% CI = 0.29–0.60). Regional anesthesia significantly reduced POD incidence in the recovery room after pediatric surgeries (0.41, 95% CI = 0.29–0.56). Regional anesthesia also reduced the POD score (SMD −0.93, 95% CI = −1.55 to −0.31) and pain score (SMD −0.95, 95% CI = −1.72 to −0.81). There was no significant difference in emergence time between regional anesthesia and general anesthesia (WMD −1.40, 95% CI = −3.83 to 6.63).

**Conclusions:**

There was a significant correlation between regional anesthesia and the decrease in POD incidence, POD score, and pain score.

## Introduction

Delirium, an acute confusion state, is characterized by reduced awareness of the environment and a disturbance in attention, which has an acute onset and fluctuating course [1]. POD is an acute and fluctuating alteration of the mental state of reduced awareness and disturbance of attention that can occur in patients of any age, from children to the elderly. It often starts in the recovery room and occurs up to 5 days after surgery [2–4]. The pathophysiological mechanisms of POD still remain unclear, while a number of important factors associated with an increased risk of delirium following surgery are universally acknowledged [5]. These included elderly, dementia and memory problems, and hearing or visual difficulties [6, 7].

For elderly patients, POD is a common surgical complication following surgery. The incidence of POD has been estimated to be up to 53% following fracture surgery in the elderly [8]. The consequences of experiencing POD include persistent cognitive impairments, poor functional recovery, higher mortality rates, hospital-acquired complications, and increased healthcare costs [9]. Moreover, POD in children is reported often. The majority of reported pediatric cases focus on emergence delirium (ED), a mental disturbance during recovery from general anesthesia, which manifests as moaning, restlessness, and involuntary physical activity, in the recovery room with a range of incidence between 2% and 80% [10–12]. This may be caused by age-related psychological issues or additional inflammatory effects on the brain, which have not been determined currently.

Despite the grave nature of POD and its associated burdens, foundational problems have tempered the pace of scientific and clinical progress. Most fundamentally, the pathophysiology of POD remains incompletely understood [13]. With an incomplete pathophysiologic understanding, a deficient diagnostic toolbox, and limited guideline evidence and implementation capacity, the prevention and management of delirium are inherently challenging. Despite the knowledge gaps in delirium pathogenesis, delirium may still be preventable with targeted, multicomponent interventions [14]. Given the harmful nature of delirium and the apparent failure of currently used drugs for prophylaxis and treatment [15], prevention efforts have expanded through the recent investigation of novel pharmacologic and non-pharmacologic strategies. Anesthetic drugs such as ketamine and dexmedetomidine might decrease the occurrence of delirium, and within the last decade, a growing body of evidence has implicated anesthetic depth as a possible contributor to POD. Thus, perioperative anesthesia may be an important intervention for POD progress which contributes to patients’ survival after surgeries.

In 2017, the European Society of Anesthesiology published guidelines on POD, while the effect of regional anesthesia on POD is controversial. It is mentioned that regional anesthesia and regional analgesia have not shown any benefit in respect of POD in the guidelines, which is based on a meta-analysis published in 2013 [16]. The evidence may be not strong, somewhat limited by the lack of studies with high quality and small sample sizes. Moreover, a number of papers have been published in the last 7 years, which may provide additional data to support or refute previous conclusions. It is also mentioned that regional anesthesia (caudal block [17, 18] and fascia iliaca compartment block [19]) is available and seems to reduce the incidence of POD. This conclusion was lack of evidence-based medical support. Based on the aforementioned limitations, this study aimed to examine the available literature works and evaluate the effect of anesthesia techniques on POD.

## Materials and methods

Initially, in this meta-analysis, addressing the intervention of anesthesia techniques was performed following the principles of the Preferred Reporting Items for Systematic Reviews and Meta-Analyses (PRISMA) statement [20, 21]. All analyses were based on previously published studies; thus, no ethical approval and patient consent are required.

### Search strategy

A search of the electronic databases Cochrane Library, Embase, and Pubmed for articles of random controlled trials published was conducted. The last retrieval was performed on July 25, 2021. The search was performed to focus on the studies reflective of modern anesthetic techniques. Search terms were applied to both subject headings and keywords and restricted to human studies without language restriction. Manual retrieval was also performed for paper documents, and the references of related reviews and included studies were further screened to obtain more appropriate studies. Search strategies can be found in Appendix 1.

### Eligibility criteria

Related studies were included based on the following criteria: (1) subjects were patients who underwent surgical operations; (2) randomized controlled trials (RCTs); (3) patients were divided into regional anesthesia and general anesthesia groups; and (4) outcomes were the risks of POD and POD score.

The exclusion criteria for this study included the following: (1) studies were involved with both regional anesthesia and other interventions (which could affect POD); (2) data could not be used for statistical analysis; and (3) studies were not RCTs.

### Study identification

The titles, abstracts, and search results were independently reviewed by two investigators (Li, Dong). The full texts of all those deemed potentially eligible were gathered and reviewed against the criteria by the same two reviewers. Full texts that met the eligibility criteria and were agreed upon by two investigators (Dai, Meng) were included. Any disagreement on study eligibility was resolved through discussion until a consensus was reached.

### Data extraction and quality assessment

Two investigators searched the literature according to the above inclusion and exclusion criteria. After eligible studies were included, the following data were extracted: the name of the first author, year of publication, ages of the subjects, types of anesthesia techniques, types of surgery, case numbers, and outcomes. Data of the first time point were extracted when these were longitudinal data. Quality assessment was conducted using the Cochrane evaluation system. The Cochrane evaluation system includes the basic contents of allocation concealment, random sequence generation, blinding of outcome assessment, blinding of participants and personnel, selective reporting, incomplete outcome data, and other biases, which can objectively and comprehensively evaluate all kinds of biases in studies. The disagreements during data extraction and quality assessment were resolved through discussion.

### Endpoints

The primary endpoint of the meta-analysis was the risk of POD incidence. The secondary endpoints were the POD score, postoperative pain score, and emergence time.

### Statistical analysis

The risk ratio (RR), mean difference (MD), weighted mean difference (WMD), and 95% confidence intervals (95% CIs) were considered as the effect sizes for calculating the merged results. The Mantel–Haenszel method was used to combine the dichotomous data, and the random variance method was used to combine the continuous data. RRs are undefined and excluded for studies with no event in either arm. For studies with zero events, 0.5 is added to the corresponding cells. A heterogeneity test was performed for the studies. When there was significant heterogeneity among the studies (*P* > 0.05, *I*^2^ > 50%), the random-effects model was applied. On the contrary, the fixed-effects model was used when homogeneous outcomes were obtained (*P* < 0.05, *I*^2^ < 50%). To evaluate the stability of the results, sensitivity analysis was conducted by removing one study each time. Subgroup analysis and regression analysis were also used to reduce heterogeneity. We assessed the possibility of publication bias by constructing a funnel plot of each trial's effect size against the standard error. Funnel plot asymmetry was assessed using the Begg and Egger tests, and significant publication bias was defined as a *P* < 0.1 [22]. Trim-and-fill computation was used to estimate the effect of publication bias on the interpretation of the results. All data analyses were performed by R version 3.33, Stata version 14.0, and RevMan version 5.3. *P* < 0.05 was considered to be statistically significant.

## Results

### Eligible studies

**Figure 1:**
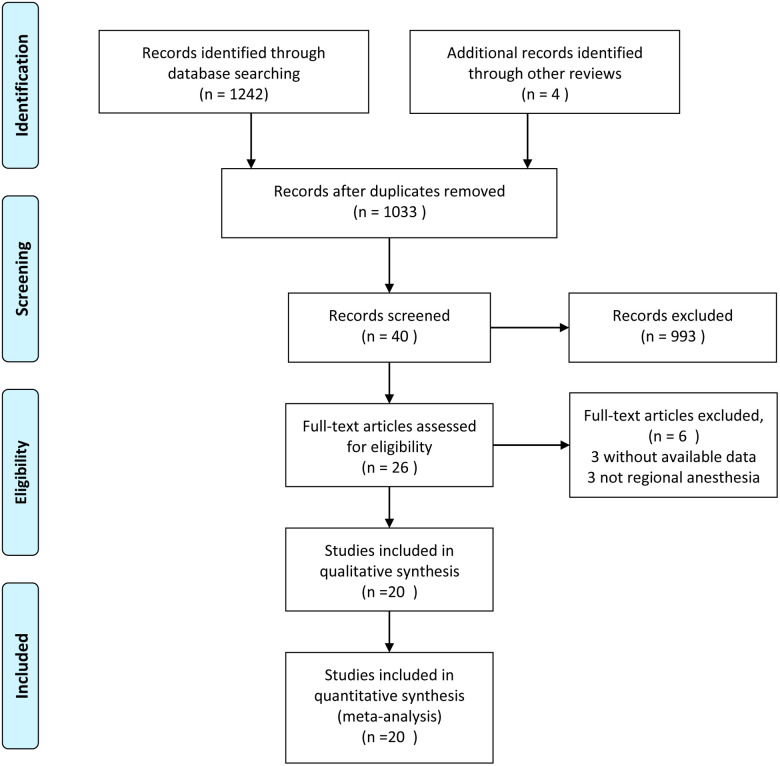
PRISMA flow chart for literature screening.

The flow chart of the article retrieval and the process of study selection is presented in Figure 1. According to the predetermined strategies, a total of 1,246 relevant studies were identified from Cochrane Library, PubMed, Embase, and citations of previous reviews. A total of 1,033 studies were saved, followed by removal of the repeated citations. After browsing the tittle, a total of 993 studies were excluded. After browsing the abstract, a total of 14 studies were excluded. Six studies were screened out following the full-text reading. Finally, a total of 20 eligible studies were selected for this meta-analysis [18, 19, 23–39].

### Study characteristics and quality assessments

**Figure 2:**
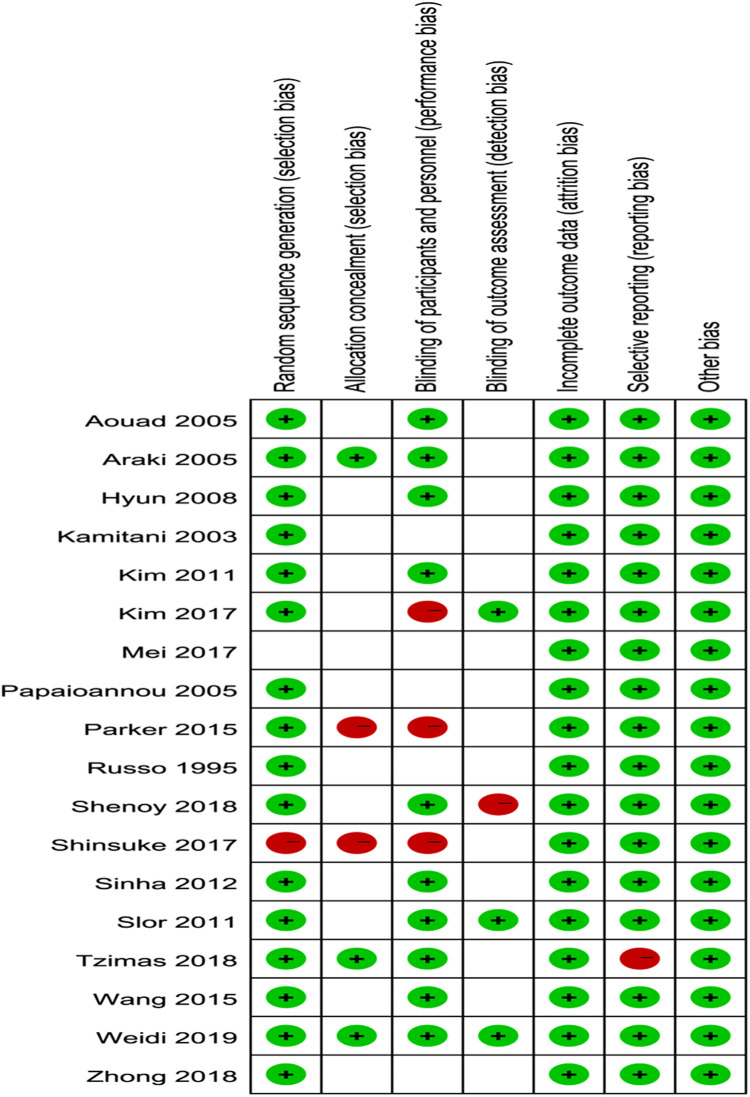
Quality assessment of the included studies.

Characteristics of the included studies are presented in Table 1. A total of 2,317 participants were included in the review. Patients in most studies were children or the elderly. According to the occurrence time, POD was divided into ED and POD (1–5 days). The most commonly used POD assessment tool was the CAM (confusion assessment method) scale and the PAED (pediatric anesthesia emergence delirium) scale. In this meta-analysis, regional anesthesia is divided into spinal anesthesia, epidural anesthesia, and nerve block. As shown in Figure 2, the methodological bias of the included studies was relatively low, indicating a high quality of the studies.

**Table 1:** Characteristics of the included studies.

Study	Country	Regional anesthesia (n); age (mean ± SD); gender (*n*)	General anesthesia (n); age (mean ± SD); gender (*n*)	Surgery type	Delirium	Follow-up	Delirium score scale	Pain score scale
Russo 1995	USA	Epidural anesthesia: lidocaine or bupivacaine + Sedation: midazolam and fentanyl (134); not reported; female (71); male (63)	Induction: thiopental sodium, fentanyl Maintenance: fentanyl, N_2_O, isoflurane (128); not reported; female (70); male (58)	Total knee replacement, elderly	POD (≥24 h)	Postoperative 7 days	Clinical features	
Kamitani 2003	Japan	Spinal anesthesia: bupivacaine (19); 83.6 ± 6; female (15); male (4)	Induction: propofol Maintenance: fentanyl, N_2_O, sevoflurane (21); 83.6 ± 6; female (21); male (0)	Artificial femoral head replacement, elderly	POD (≥24 h)	Postoperative 4 days	Not reported	
Araki 2005	Japan	Caudal block: bupivacaine + general anesthesia: Before induction: midazolam Induction: N_2_O, sevoflurane Maintenance: N_2_O, sevoflurane (15); 3.4 ± 1.9; female (4); male (11)	Before induction: midazolam Induction: N_2_O, sevoflurane Maintenance: N_2_O, sevoflurane (15); 2.5 ± 1.8; female (7); male (8)	Herniorrhaphy, children	ED	PACU period	three-point agitation scale	
Aouad 2005	USA	Caudal anesthesia: plain racemic bupivacaine + general anesthesia: Before induction: midazolam Induction: sevoflurane Maintenance: sevoflurane (22); 4 ± 1.6; female (4); male (18)	Before induction: midazolam Induction: sevoflurane Maintenance: fentanyl, sevoflurane (22); 3.9 ± 1.5; female (4); male (18)	Herniorrhaphy, children	ED	PACU 30 min	four-point agitation scale	
Papaioannou 2005	Greece	Spinal or epidural anesthesia + sedation (19); not reported; female (7); male (12)	General anesthesia (28); not reported; female (10); male (18)	Abdominal surgeries, elderly	POD (≥24 h)	Postoperative 3 days	DSM III criteria	
Hyun 2008	Korea	Skull block: bupivacaine + general anesthesia: Induction: thiopental sodium Maintenance: N_2_O, sevoflurane (18); 8.8 ± 2.5; female (12); male (6)	Induction: thiopental sodium Maintenance: N_2_O, sevoflurane (21); 7.8 ± 2.9; female (16); male (5)	EDAMS surgery, children	ED	PACU period	Five-point agitation scale	VAS
Slor 2011	Netherlands	Spinal anesthesia (337); 78.2 ± 6; female (262); male (67)	General anesthesia (189); 76.7 ± 5.5; female (148); male (45)	Hip surgery, elderly	POD (≥24 h)	Postoperative 5 days	DSM IV criteria and CAM	
Kim 2011	Korea	Fascia iliaca compartment block: ropivacaine + general anesthesia: Induction: N_2_O, sevoflurane Maintenance: N_2_O, sevoflurane (32); 4.75 ± 1.5; female (15); male (17)	Induction: N_2_O, sevoflurane Maintenance: N_2_O, sevoflurane (32); 4.5 ± 1.8; female (18); male (14)	Orthopedic surgery, children	ED	PACU 30 min	PAED scale	CHEOPS
Sinha 2012	India	Caudal block: bupivacaine + general anesthesia : Before induction: midazolam, Induction: N_2_O, sevoflurane Group BK: Maintenance: N_2_O, sevoflurane (60); 3.18 ± 1.66; female (11); male (49) Group B: Maintenance: ketamine, N_2_O, sevoflurane(60); 2.88 ± 1.48; female (8); male (52)	Before induction: midazolam Induction: N_2_O, sevoflurane Maintenance: fentanyl, N_2_O, sevoflurane (60); 2.85 ± 0.95; female (9); male (51)	Herniorrhaphy, children	ED	PACU period	PAED scale	OPS
Parker 2015	United Kingdom	Spinal anesthesia (158); 82.9; female (128); male (30)	General anesthesia (164); 83; female (107); male (57)	Hip fracture surgery	POD (≥24 h)	Not available	Ten-question mental test	
Wang 2015	China	Infraorbital nerve block: bupivacaine + general anesthesia: Induction: fentanyl, N_2_O, sevoflurane Maintenance: dexamethasone, N_2_O, sevoflurane (50); 3.3 ± 2.0; female (15); male (15)	Induction: fentanyl, N_2_O, sevoflurane Maintenance: dexamethasone, N_2_O, sevoflurane (50); 3.1 ± 2.1; female (13); male (17)	Cleft lip surgery, children	ED	PACU	PAED scale and Five-point agitation scale	CHIPPS
Glover 2016	USA	supraclavicular block + general anesthesia; not reported; not reported	General anesthesia; not reported; not reported	Percutaneous pinning, children	ED	PACU period	PAED scale	FLACC and verbal pain scale
Mei 2017	China	Lumbosacral plexus block: ropivacaine + general anesthesia: Induction: sufentanil, propofol Group D: Maintenance: propofol; Bis: 40–60 (66); 75 ± 6; female (38); male (28); Group L: Maintenance: propofol, Bis: 60–80 (66); 77 ± 8; female (42); male (24)	Group G: Induction: sufentanil, propofol Maintenance: sufentanil, propofol; Bis 40–60 (66); 77 ± 8; female (29); male (37)	Total hip arthroplasty, elderly	POD (≥24 h)	Postoperative 3 days	CAM	VAS
Kim 2017	Korea	Scalp nerve block: ropivacaine + general anesthesia: Induction: thiopental sodium, sevoflurane, Maintenance: sevoflurane (22); 3.4 ± 2.1; female (12); male (10)	Induction: thiopental sodium, sevoflurane Maintenance: remifentanil, sevoflurane (22); 2.9 ± 1.6; female (12); male (10)	Nevus surgery, children	ED	PACU 30 min	Watcha scale	FLACC
Shinsuke 2017	Japan	Femoral nerve and sciatic nerve with/without obturator nerve block (31)	General anesthesia (31)	Infrapopliteal artery bypass grafting	POD (≥24 h)	Postoperative 30 days	Not reported	
Zhong 2018	China	Fascia iliaca compartment block: ropivacaine + general anesthesia: Before induction: midazolam, Induction: fentanyl, propofol, Maintenance: N_2_O, sevoflurane (40); 8.7 ± 2.2; female (12); male (28)	Before induction: midazolam Induction: fentanyl, propofol Maintenance: N_2_O, sevoflurane (40); 8.4 ± 1.7; female (16); male (24)	Femoral fracture surgery, children	ED	PACU	Not reported	
Tzimas 2018	Greece	Spinal anesthesia: fentanyl, ropivacaine (37); 77.11 ± 6.5; not reported	Induction: fentanyl, propofol Maintenance: desflurane (33); 75.09 ± 6.08; not reported	Hip fracture surgery, elderly	POD (≥24 h)	Postoperative 30 days	CAM	
Shenoy 2018	India	Transversus abdominis plane: ropivacaine + general anesthesia: Before induction: midazolam, glycopyrrolate Induction: fentanyl, propofol, Maintenance: N_2_O, sevoflurane (71); 10.9 ± 5.4; not reported	Before induction: midazolam, glycopyrrolate Induction: fentanyl, propofol Maintenance: N_2_O, sevoflurane (72); 10.6 ± 6.4; not reported	Iliac crest bone graft harvesting	ED	Postoperative 60 min	Watcha scale	FLACC
Weidi 2019	China	Group RD: retrobulbar block: ropivacaine, dexamethasone (40); 4.5 ± 1.2; female (24); male (16) Group RB: retrobulbar block: ropivacaine (40); 4.4 ± 1.6; female (19); male (21)	Group F: Induction: propofol, remifentanil Maintenance: propofol, remifentanil (40); 4.2 ± 2.0; female (17); male (23)	Vitreoretinal surgery, children	ED	Postoperative 120 min	PAED scale	FLACC

### Synthesis of results

#### Meta-analysis of the primary endpoint

**Figure 3:**
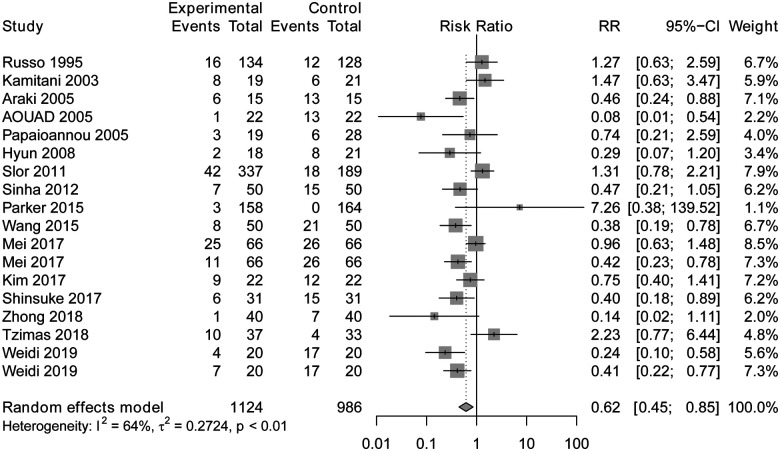
Forest plot of regional anesthesia on POD incidence.

**Figure 4:**
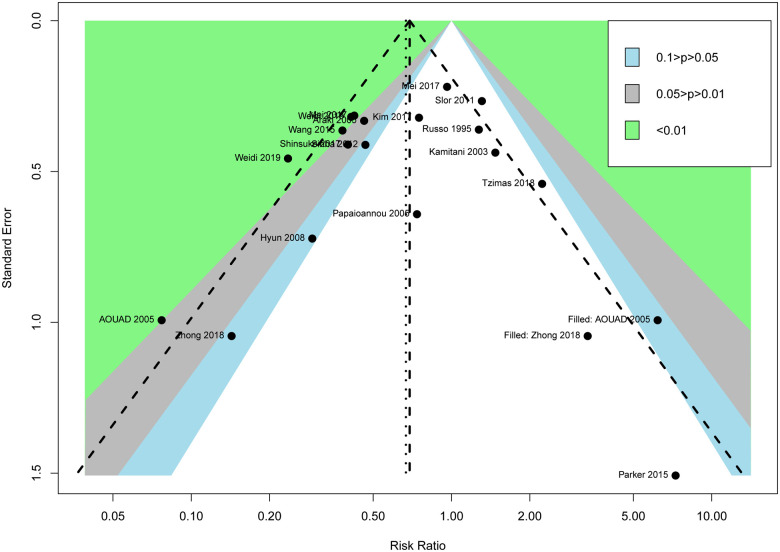
Contour-enhanced funnel.

Regional anesthesia was significantly associated with decreased POD incidence compared to general anesthesia (RR 0.62, CI: 0.45–0.85, *P* < 0.01) according to the random-effects model (Figure 3). Meta-analysis revealed that regional anesthesia significantly reduced the risk of POD incidence by 38%, with a relative risk of 62%. The sensitivity analyses of POD incidence showed the result was stable. The Begg and Egger tests indicated that the funnel plot was asymmetric. The contour-enhanced funnel showed that it is necessary to include two articles with no statistical difference to achieve symmetry, which indicated that the asymmetry originated from publication bias (Figure 4).

#### Meta-analysis of secondary endpoints

**Figure 5:**
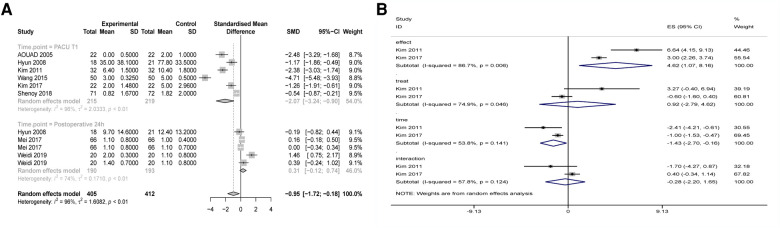
(**A**) Forest plot of regional anesthesia on POD score. (**B**) Forest plot of multilevel analysis on POD score.

**Figure 6:**
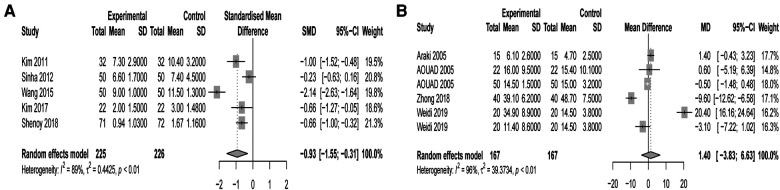
(**A**) Forest plot of regional anesthesia on postoperative pain score. (**B**) Forest plot of regional anesthesia on emergence time.

The SMD of the POD score was significantly different (SMD −0.93, 95% CI: −1.55 to −0.31) according to the random-effects model (Figure 5A). Regional anesthesia reduced 0.93 points of the POD score compared with general anesthesia. As POD scores and observation time points in PACU were longitudinal data, a multilevel analysis was conducted. Merged results showed significantly different POD scores between different time points (postoperative 0, 10, and 20 min) and no significantly different POD scores between regional and general anesthesia (Figure 5B). Regional anesthesia reduced the postoperative pain score (SMD −0.95, 95% CI: −1.72 to −0.81), and subgroup analysis showed that regional anesthesia reduced the pain score within PACU time (SMD −2.07, 95% CI: −3.24 to −0.90) rather than postoperative 24 h (SMD 0.31, 95% CI: −0.12 to 0.74) (Figure 6A). Four studies showed no significant difference in emergence time (WMD −1.40, 95% CI: −3.83 to 6.63) (Figure 6B).

#### Subgroup analysis

**Figure 7:**
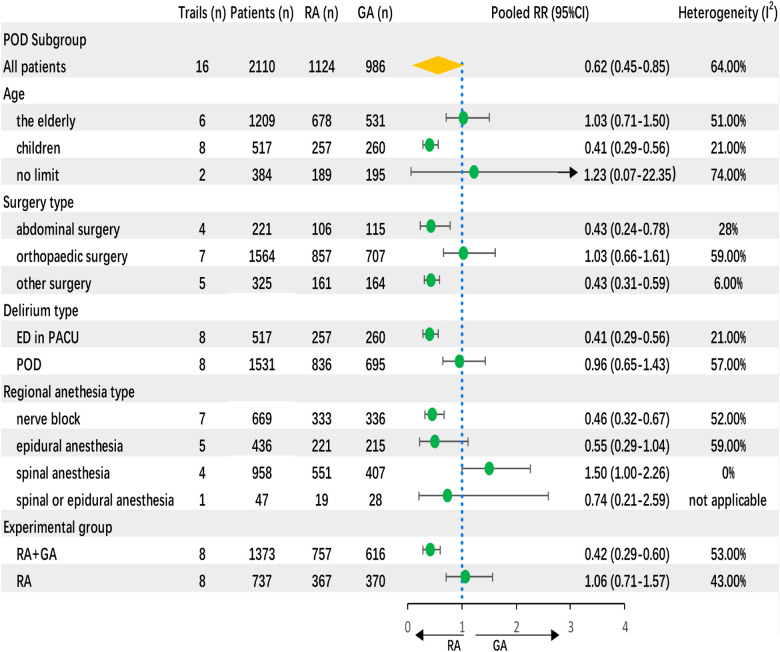
Funnel plot of subgroup analysis on POD incidence.

Four subgroups were established based on characteristics of patients. Based on age of participants, regional anesthesia decreased POD incidence of children (0.41, 95% CI: 0.29 to 0.56) rather than the elderly (1.03, 95% CI: 0.71–1.50). Based on surgery types, regional anesthesia decreased the POD incidence in abdominal surgery (0.43, 95% CI: 0.24–0.78) instead of orthopedic surgery (1.03, 95% CI: 0.66–1.61). Based on onset time, regional anesthesia decreased ED (0.41, 95% CI: 0.29–0.56) but not POD (1–5 days) (0.96, 95% CI: 0.65–1.43). Based on regional anesthesia techniques, nerve block (0.46, 95% CI: 0.32–0.67) decreased POD incidence instead of epidural (0.55, 95% CI: 0.29–1.04) and spinal anesthesia (1.5, 95% CI: 1–2.26). Also, regional-combined-general anesthesia decreased the POD incidence significantly (0.42, 95% CI: 0.29–0.60) (Figure 7).

## Discussion

After delirium episodes, the postepisode intervention has little effect on severity or duration. However, delirium is able to be prevented before its onset, which emphasizes the importance of primary prevention [40, 41]. Also, this can be achieved by interventions tackling risk factors, such as adequate pain management, hearing or visual aid, sleep enhancement, exercise training, and dietary advice [42, 43]. Based on the analysis of evidence-based medical support, a number of measures have been suggested and applied perioperatively to reduce POD. In order to reduce the incidence of POD by controlling anesthetic techniques, the association between regional anesthesia and POD was fully analyzed in the current meta-analysis.

### Explanation of results

Previous studies support the use of regional anesthetics in improving postoperative cognitive complications [44, 45]. This conclusion originated from a cohort study and systematic review. An RCT demonstrated that peripheral lumbosacral plexus block has beneficial effects on POD in elderly patients receiving total hip arthroplasty. In this meta-analysis, a total of 21 RCTs were included according to the eligible criteria, of which 20 were selected for analysis. The quality assessment showed that the selected RCTs had high qualities. The results indicated that regional anesthesia could decrease POD incidence. The sensitivity analysis showed that the merged result of POD incidence was stable. We predict the following reasons for this result: (1) regional anesthesia is commonly accompanied by a low depth of sedation. In 2010, Sieber et al. investigated the influence of sedation on POD incidence in patients undergoing hip fracture repair surgery. They found that deep sedation was associated with a high incidence of POD [46]. Another study demonstrated that intraoperative electroencephalogram suppression is an independent risk factor for POD [47]. A study published in 2018 demonstrated that under lower volatile anesthetic concentration, intraoperative electroencephalogram suppression could predict occurring of POD [48]. The current results may be derived from a low sedation depth of regional anesthesia, although the mechanisms of sedation depth in impairing postoperative cognitive function are complicated and have not been investigated thoroughly. (2) Compared with general anesthesia, nerve block contributed to stable hemodynamics. The unstable hemodynamics could influence the perfusion of cerebrovascular. Poorer cerebral perfusion pressure is associated with greater risk for POD, as well as longer duration and severity of delirium, and poor cerebral perfusion is an independent risk factor of POD [49]. Recently, an RCT published in JAMA Surgery demonstrated that optimizing mean arterial pressure to be greater than the individual patient's lower limit of cerebral autoregulation contributes to reducing POD incidence [50]. (3) Regional anesthesia provides effective postoperative analgesia, which contributes to the reduction of POD incidence. An important factor in managing POD is adequate stress reduction with sufficient analgesia, an appropriate choice of analgesia [51]. Using a continuous intraoperative analgesia regimen might reduce the incidence of POD.

Subgroup analysis demonstrated that regional anesthesia decreased the POD incidence of children in PACU (ED) but had no effect on POD (postoperative 1–5 days) of elderly patients. Meta-analysis of the secondary endpoints may help to explain this finding. The merged results showed that regional anesthesia reduced 0.93 average points of POD score of the first observation time point in PACU compared with general anesthesia. Also, regional anesthesia reduced the pain score within PACU time rather than postoperative 24 h. Continuous analgesia in PACU contributes to the incidence of POD and the POD score. Results of the multilevel analysis showed significantly different POD scores between different time points (postoperative 0, 10 and 20 min) and no significant different POD scores between regional and general anesthesia. It means that the decrease in POD score over time was not related to regional anesthesia and time is the independent risk factor.

It should be noted that conduction anesthesia decreased the POD incidence rather than epidural and spinal anesthesia. The reason may be that nerve block contributes to stable hemodynamics and provides effective postoperative analgesia. The mechanisms have been described above. Interestingly, regional anesthesia decreased the POD incidence in abdominal surgery rather than orthopedic surgery. The accuracy and mechanism of this finding need to be further studied.

### Clinical significance of the current finding

The current management approach to POD is mainly focused on the prevention of delirium. However, if the precipitating factor is surgery, which is inevitable, other perioperative approaches should be considered. Managing the perioperative predisposing factors of delirium is vital and would decrease the morbidity and mortality associated with POD. A recent study used topological data analysis (TDA) to assess phenotypic subgroups of delirium and indicated that regional anesthesia was one of the predictive risk factors of POD [52]. Results of this meta-analysis showed that regional anesthesia decreased the POD incidence, which confirmed the conclusions of the previous research. Subgroup analysis demonstrated that regional anesthesia decreased the ED incidence of children in PACU but had no effect on POD (postoperative 1–5 days) of elderly patients and time is the independent risk factor. This may indicate that regional anesthesia may be an important measure for reducing early delirium postoperatively. All these findings may have clinical significance in the prevention of POD.

### Limitations

The main limitation of this meta-analysis is the small sample size of a few included studies. Also, publication bias was found for POD incidence. The contour-enhanced funnel showed that it is necessary to include two articles with no statistical difference to achieve symmetry. In addition, the present analysis could not get enough data from all the included studies. One study was published as an abstract, and the relevant data could not be extracted. Some unpublished data on ongoing RCTs also could not be obtained.

## Conclusions

In conclusion, our results showed that regional anesthesia significantly reduced the POD incidence and POD score. This effect of regional anesthesia is especially reflected in children during PACU time rather than elderly patients during postoperative 1–5 days. Since the guidelines have not provided strong evidence of regional anesthesia on POD, to some extent, our results are complementary to the guidelines. However, more studies with large sample sizes are still needed to support the present results.

## Data Availability

The original contributions presented in the study are included in the article/Suplementary Material; further inquiries can be directed to the corresponding author/s.

## Appendix 1 Pubmed Search strategy based on PICOS

1. Anesthesia, Intravenous [MeSH] OR text word: Anesthesias, Intravenous; Intravenous Anesthesia; Intravenous Anesthesias
2. Anesthesia, Local [MeSH] OR text word: Local Anesthesia; Anesthesia, Infiltration; Infiltration Anesthesia; Neural Therapy of Huneke; Huneke NeuralTherapy
3. Anesthesia, Endotracheal [MeSH] OR text word: Anesthesias, Endotracheal; Endotracheal Anesthesias; Intratracheal Anesthesia; Anesthesias, Intratracheal; Intratracheal Anesthesias; Anesthesia, Intratracheal
4. Anesthesia, Inhalation [MeSH] OR text word: Inhalation Anesthesia; Insufflation Anesthesia; Anesthesia, Insufflation
5. General [MeSH]; text word: Anesthesias, General; General Anesthesia; General Anesthesias
6. Anesthesia, Epidural [MeSH] OR text word: Anesthesia, Peridural; Anesthesias, Peridural; Peridural Anesthesia; Peridural Anesthesias; Anesthesia, Extradural; Anesthesias, Extradural; Extradural Anesthesia; Extradural Anesthesias; Epidural Anesthesia; Anesthesias, Epidural; Epidural Anesthesias
7. Anesthesia, Conduction [MeSH] OR text word: Conduction Anesthesia; Anesthesia, Regional; Regional Anesthesia
8. Nerve Block [MeSH] OR text word: Block, Nerve; Blocks, Nerve; Nerve Blocks; Nerve Blockade; Blockade, Nerve; Blockades, Nerve; Nerve Blockades; Chemical Neurolysis; Chemical Neurolyses; Neurolyses, Chemical; Neurolysis, Chemical; Chemodenervation; Chemodenervations
9. neuraxial anesthesia OR anesthetic technique
10. Delirium [MeSH] OR postoperative delirium OR POD OR emergence delirium OR emergence agitation OR mortality
11. Randomized Controlled Trial [Mesh] OR RCT OR RCTs OR randomized
12. (1 OR 2 OR 3 OR 4 OR 5 OR 6 OR 7 OR 8 OR 9) AND 10 AND 11
